# ALTERED TRYPTOPHAN DEGRADATION LINKS CHRONIC INFLAMMATION TO FUNCTIONAL DECLINE & FRAILTY IN MICE AND HUMANS

**DOI:** 10.1093/geroni/igz038.3473

**Published:** 2019-11-08

**Authors:** Reyhan Westbrook, Tae Chung, Jacqueline Lovett, Chris Ward, Mohammed Khadeer, Ruin Moaddel, Jeremy Walston, Peter Abadir

**Affiliations:** 1 Division of Geriatric Medicine and Gerontology, Johns Hopkins University School of Medicine, Baltimore, Maryland, United States; 2 Department of Physical Medicine and Rehabilitation, Johns Hopkins University School of Medicine, Baltimore, Maryland, United States; 3 National Institute on Aging, National Institutes of Health, Baltimore, Maryland, United States; 4 Department of Orthopedics, University of Maryland School of Medicine, Baltimore, Maryland, United States; 5 Johns Hopkins University School of Medicine, Baltimore, Maryland, United States

## Abstract

Chronic inflammation is associated with frailty and functional decline in older adults but the molecular mechanisms of this linkage are not well understood. We sought to examine metabolic and physiologic states associated with aging and frailty by analyzing the composition of metabolites in the blood of a population of community dwelling young, and older adults. Serum inflammatory cytokines and demographic and physiological covariates were collected in a set of community-dwelling adults age 20-97 (n=166). We then used LC/MS technology to profile 121 metabolites from five substance classes. Associations of the cytokines and metabolites with grip strength, walking speed, falls and outcomes were assessed in young, robust, pre-frail and frail participants. Age and frailty status positively correlated with IL6, TNFα, TNFαR1, IL1β (p<0.0001). Analysis of metabolites revealed significant alterations in tryptophan degradation pathway with aging and frailty. Among the top metabolites to correlate with age and frailty status were kynurenine (p<0.0001) and the kynurenine/tryptophan ratio (p<0.0001). The kynurenine/tryptophan ratio also tightly correlated with serum inflammatory cytokines TNFαR1 (p<0.0001) and IL-6 (p<0.0001). Higher kynurenine/tryptophan levels were associated with weaker grip strength and slower walking speed, even after adjusting for age, gender, BMI and blood pressure. Further dissection of the pathway revealed the accumulation of 3-hydroxykynurenine, a cytotoxic and neurotoxic intermediate from the kynurenine pathway, with frailty. The increased levels of cytotoxic and neurotoxic molecules in this pathway may in part explain the link between inflammation and cognitive and physical decline in frailty.

